# Long-Term Outcomes of Children Receiving Percutaneous Endoscopic Gastrostomy Feeding

**DOI:** 10.3390/medicina61030366

**Published:** 2025-02-20

**Authors:** Mahmood Grayeb, Avishay Lahad, Rana Elhaj, Marwan Elias, Yael Shmaya, Firas Rinawi

**Affiliations:** 1 The Azrieli Faculty of Medicine, Bar-Ilan University, 8 Henrietta Szold St, POB 1589, Safed 1311502, Israel; 2 Pediatric Gastroenterology Unit, Emek Medical Centre, Afula 1834111, Israel; avishay_la@clalit.org.il (A.L.); rana.elhaj@clalit.org.il (R.E.); yael.shmaya@gmail.com (Y.S.); firasytr@gmail.com (F.R.); 3 Pediatric Surgery Department, Emek Medical Centre, Afula 1834111, Israel; eliasmmd@gmail.com; 4 Faculty of Medicine, Technion, 1 Efron St. Bat Galim 3525433, Haifa 3109601, Israel

**Keywords:** children, neurological, developmental delay, PEG, vitamin D, iron

## Abstract

*Background and Objectives*: Data regarding long-term outcomes of gastrostomy-fed children is scarce. The aim of the study was to analyze the long-term follow-up of children receiving percutaneous endoscopic gastrostomy (PEG) in terms of nutritional outcomes, hospitalization, and fundoplication rates. *Materials and Methods*: The medical records of gastrostomy-fed children who underwent PEG placement between January 2002 and June 2022 and subsequently attended primary care clinics of the Clalit Health Services (CHS) in Northeastern Israel, were reviewed in this retrospective cohort study. *Results*: A total of 372 gastrostomy tubes (GT) were placed, 88% of the children had neuro-developmental impairment. During the median follow-up of 64 months, 230 patients (62%) had frequent recurrent hospitalizations defined as at least two hospitalizations per year on average. Hospitalizations were due to respiratory infections in 52%. Among 322 patients who underwent iron status work-up, (64%) and (31%) had iron deficiency (ID) and ID anemia, respectively. Laboratory monitoring of other micronutrient levels was limited but showed that 25/73 (34%) had vitamin D deficiencies, without significant association with recurrent hospitalization (*p* > 0.1). A total of 12% of the patients underwent subsequent fundoplication. *Conclusions*: This study confirmed the durability of gastrostomy tube feeding in children with neurological impairment, noting a low prevalence of fundoplication but a high rate of hospitalizations, primarily due to respiratory infections. Regular assessment of micronutrient deficiencies, particularly vitamin D, is recommended for these patients.

## 1. Introduction

Pediatric patients with chronic diseases, including neurodevelopmental delay, metabolic disorders, or cerebral palsy (CP), often experience growth delays and restrictions in their nutritional status due to feeding issues, high nutritional requirements, and/or inadequate caloric intake. Such conditions necessitate specific nutritional support to prevent undernutrition and growth failure [1,2,3,4,5]. Home enteral nutrition, particularly via gastrostomy tube (GT), has been proven to be a secure technique for these patients, improving or reversing undernourishment, simplifying care, reducing hospitalization lengths, and decreasing respiratory and infectious complications [6,7,8,9]. Percutaneous endoscopic gastrostomy (PEG) feeding is a procedure in which a feeding tube is placed directly into the stomach through the abdominal wall using endoscopic guidance. This method is primarily used for patients who have a functional gastrointestinal system but are unable to meet their nutritional needs through oral intake. PEG is a minimally invasive procedure used to provide long-term enteral nutrition for patients with impaired oral intake [10]. Compared to surgical insertion, PEG is associated with potential advantages such as lower complication rates, shorter recovery periods, reduced costs, and improved patient tolerance [10]. These factors support PEG’s inclusion in clinical guidelines [11]. A high proportion of gastrostomy-fed children with neurodevelopmental delays develop gastrointestinal (GI) symptoms, including constipation, GI dysmotility, and gastro-esophageal reflux disease (GERD), which might contribute to abdominal discomfort and decreased caloric intake even via GT [12]. This necessitates performing subsequent fundoplication in order to improve enteral intake [13].

Pediatric formulas are used to avoid micronutrient deficiencies [14,15]. However, data regarding the prevalence of nutritional deficiencies, anthropometric measurements, and long-term nutritional status among gastrostomy-fed children is scarce [16,17,18]. Studies also suggest some micronutrients, such as iron and zinc, are essential for maintaining a functional immune response, especially in children receiving long-term enteral nutrition [19]. Vitamin D deficiency may be relatively common among gastrostomy-fed children, particularly those with neurodevelopmental delay and decreased sun exposure, as well as those with reduced formula intake due to gastrointestinal intolerance or who are not on exclusive GT feeding. In addition, vitamin D plays a vital role in immune system modulation, contributing to the prevention of infections, particularly respiratory tract infections, in vulnerable populations [20]. Vitamin D has antioxidant, anti-inflammatory, and immune-modulatory properties of both the innate and adaptive immune responses by enhancing the production of antimicrobial peptides like cathelicidin, which combat infections [21]. This role of vitamin D is particularly important in children with neurological disorders, who are already at higher risk for respiratory infections due to their compromised health status [22,23]. The association between low vitamin D levels and respiratory infections, such as community-acquired pneumonia and sinusitis, has been demonstrated in adults without neurological disorders [24,25]. To our knowledge, no studies have investigated such an association among gastrostomy-fed children with neurodevelopmental delay. The aim of the present study was to evaluate the long-term follow-up of children receiving GT feeding in terms of hospitalization, nutritional outcomes, and fundoplication rates.

## 2. Materials and Methods

This is a retrospective cohort study of Clalit Health Services (CHS) members, the largest of 4 integrated healthcare organizations in Israel, including 4.7 million members (53% of the population) [26]. The medical records of gastrostomy-fed children who underwent PEG placement (age: 0–18 years at the time of tube insertion) between January 2002 and June 2022 and subsequently were attending primary care clinics of the CHS in Northeastern Israel were retrospectively reviewed for child age at the time of tube placement, weight z-score, micronutrient deficiencies, GI symptoms, medications, hospitalization and surgical intervention reasons, and the number of hospitalizations. Patients were identified using ICD-9 codes from the Health Maintenance Organization (HMO)’s administrative data. Patients with a history of inflammatory bowel disease, malignancy, allergic gastroenteropathy, eosinophilic gastroenteritis, cystic fibrosis, or other disorder of malabsorption and patients who underwent primary surgical gastrostomy tube placement, primary gastrojejunostomy (GJ) tube, or jejunostomy tube were excluded. Our outcomes included developing micronutrient deficiencies such as iron, vitamin D, folic acid, or vitamin B12. Frequent recurrent hospitalizations were defined as an average of at least two hospital admissions per year, with a minimum interval of two weeks between each hospitalization episode. In this study, we followed the guidelines of the World Health Organization (WHO) and the American Academy of Pediatrics (AAP), which have defined ID as ferritin levels below 12 and 15 μg/L in otherwise healthy children under and above 5 years, respectively. Anemia was defined by a reduced Hb value 5th percentile below the normal Hgb value specified for age and sex [25]. Vitamin D deficiency was defined as a serum 25-hydroxyvitamin D [25(OH)D] level of less than 20 ng/mL [26].

In addition, patient comorbidities were reviewed and categorized into the following categories: neurologic, metabolic/genetic, cardiac, oncologic, or oropharyngeal abnormalities. Comorbidities were not considered to be mutually exclusive; therefore, some patients could have >1 comorbidity listed. Primary indications for PEG tube placement were documented and included poor weight gain, aspiration (defined as having a documented abnormal modified barium swallow before PEG placement), or other feeding difficulties (excluding any patients with documented aspiration on modified barium swallow). In addition, we collected data regarding chronic or recurrent GI or respiratory symptoms, pharmacological treatments, surgical interventions (fundoplication), and death. Data regarding pharmacological treatments were available only for prescribed medications, so the proportion of patients treated with osmotic laxatives or iron supplements could not be assessed accurately since a relatively high percentage of them were probably treated without prescriptions.

Statistical Analysis: Data were analyzed using SPSS (version 21.0, SPSS, Inc., Chicago, IL, USA). Continuous variables were presented as either mean ± SD or median with interquartile range (IQR), depending on the data approximation to normal distribution. To analyze the risk factors predictively, Fisher’s exact test was used to explore univariate associations between primary outcomes and categorical variables. Associations of continuous variables with primary outcome measures were examined using ANOVA with repeated measures. Associations between patients’ characteristics and risk of fundoplication or recurrent hospitalization due to respiratory infection were evaluated using uni and multivariate Cox regression. All reported *p*-values are two-sided. *p*-values < 0.05 is considered significant. Variables with *p*-value < 0.2 were included in the multivariate Cox regression analysis. The study was approved by the local IRBs at the Emek Medical Center and at the Northern District of Clalit Health Services.

## 3. Results

Among children born between January 2002 and June 2022 in northeastern Israel, the reported prevalence of new gastrostomy tube insertion was 14 cases per 10,000 live births. In total, we had 412 percutaneous endoscopic gastrostomy tubes (GT), which were placed in total between January 2002 and June 2022: 20 patients were excluded, and data were not available for an additional 20 children. In our cohort of 372 patient’s, the median age at GT insertion was 22.8 months (IQR 11.5–47.2). Two hundred and one were males (54%). No gastrostomy insertion-related death was documented. During follow-up, death under the age of 18 years was documented in 94 patients (26%), and the median time from insertion to death was 26 months (IQR 12–67). The most frequent underlying diagnosis for GT insertion was neurodevelopmental delay (88%), while 82 patients (22%) had seizures (Table 1).

Among 372 children, the principal symptoms leading to gastrostomy insertion were feeding difficulties (74%), recurrent vomiting (53%), failure to thrive (38%), and recurrent aspiration (29%) (Table 1). The median length of follow-up was 64 months (IQR 28–106). The most common prolonged prescribed medications were osmotic laxatives in 212 patients (57%), proton pump inhibitors (53%), inhaled b-blockers/corticosteroids (50%), and prophylactic antibiotics (13%). Among 322 patients who underwent iron status work-up, 238 (64%) and 100 (31%) had iron deficiency ID and ID anemia, respectively. Of 372 children, during the follow-up period, except for iron status, laboratory work-up for other micronutrient deficiencies was limited; 8/78 (10%), 4/69 (6%), and 25/73 (34%) had vitamin B12, folic acid, and vitamin D deficiencies, necessitating supplementation.

The median weight z-score at the time of PEG insertion was (−1.8), IQR (−2.4)–(−0.9), while at the end of follow-up, it increased to (−1.65), IQR (−2.1)–(−0.5), (*p* = 0.004). During follow-up, 230 patients (62%) had frequent recurrent hospitalizations. The majority of the hospitalizations were due to respiratory infections (52%), convulsions or other neurological symptoms (44%), GI symptoms (27%), or non-GI and non-respiratory infections (21%). In the multivariate Cox model analysis (Table 2), sustained treatment with PPIs or inhaled b-blockers/corticosteroids was significantly associated with recurrent hospitalization due to respiratory infections (HR 1.88, 95% CI 1.46–3.07, *p* = 0.033 and HR 1.45, 95% CI 1.28–3.25, *p* = 0.003, respectively). In addition, the history of seizures increased the risk for these recurrent hospitalizations (HR 1.502, 95% CI 1.302–6.642, *p* = 0.014). Other factors that significantly predict recurrent hospitalizations during follow-up in the univariate Cox proportional hazard models (male gender, younger age at GT insertion, continuous antibiotic prophylaxis, and frequent recurrent hospitalizations during follow-up) did not maintain their significance in the multivariate analysis (Table 2).

Forty-five children (12%) underwent fundoplication, the median time from insertion to fundoplication being 32 months (IQR 16–44); all of them were neurologically impaired. In the multivariate Cox model analysis (Table 3), sustained treatment with PPIs or inhaled b-blockers/corticosteroids was significantly associated with fundoplication (HR 1.95, 95% CI 1.66–3.76, *p* < 0.001 and HR 1.25, 95% CI 1.1–3.05, *p* = 0.041, respectively). Among the 45 children who underwent fundoplication, 28 (62%) continued PPI treatment, and 9 (20%) required a second anti-reflux procedure following the initial fundoplication. Death during childhood was documented in 94 patients (25%); the median time from insertion to death was 26 months (IQR 12–67).

## 4. Discussion

In the current study, the prevalence of gastrostomy tube insertion was elevated compared to the findings from previous research [27,28]. This disparity may be attributed to a higher regional burden of neurodevelopmental delays and metabolic disorders, potentially linked to a greater prevalence of consanguinity. In this study, we examined the clinical and nutritional outcomes of gastrostomy-fed children. Consistent with a previous study [29] that demonstrated that two-thirds of children requiring GT insertion were diagnosed with CP, our cohort predominantly comprised children diagnosed with neurodevelopmental delay.

Our results indicated a lack of proper follow-up and assessment of micronutrients in this population, except for iron. Despite the adequate follow-up of iron levels, a high percentage of ID and ID anemia were observed. From the data collected over 20 years regarding the tube-fed pediatric population, it was evident that iron was the only micronutrient monitored frequently in the majority of children. These findings are in accordance with a previous study by Le Roy et al. (2021) [18], which revealed that more than half of children and adolescents diagnosed with CP had low ferritin levels. This could potentially be explained by the partial (not exclusive) feeding with iron-enriched formulas or the avoidance of oral iron treatments due to concerns about worsening constipation, which is prevalent in this population. Among anthropometric measurements, weight was the only parameter monitored and followed. We observed a significant improvement in weight z-scores during follow-up, indicating adequate caloric intake in the majority of patients. A study from 2009 [3] demonstrated that poor energy intake could exacerbate motor disabilities, highlighting the importance of proper nutrient monitoring in this pediatric group.

More clinical follow-up is necessary to determine the impact of poor micronutrient status in these children, making sufficient nutritional follow-up a high priority, particularly in children with motor disabilities. Except for iron, other micronutrients were seldom checked among the minority of children who were checked for vitamin D levels (only a fifth of the cohort), a significant portion (approximately one-third) had a deficiency in this micronutrient. Our findings indicated a relatively higher prevalence of vitamin D deficiency compared to published studies. For instance, an Australian study conducted in 2003 [30] reported that 25% of chronically ill or disabled children had a vitamin D deficiency. On the other hand, a study from 1999 to 2002 reported no vitamin D deficiency in children with gastrostomy or jejunostomy [17]. The relatively higher prevalence of vitamin D deficiency in our cohort could be attributed to selection bias, where physicians might check vitamin D levels when there is a high clinical suspicion due to symptoms. Other explanations included a higher proportion of patients with reduced sun exposure, or insufficient vitamin D intake particularly those who were on partial GT feeding of polymeric or elemental formulas. The paucity of data on micronutrient deficiencies, beyond iron, necessitates a cautious interpretation of these results. Future investigations should emphasize comprehensive micronutrient profiling to enhance clinical insights and optimize patient management strategies.

The association between vitamin D deficiency and susceptibility to infections has been described in the literature [24,31]. Despite that, no such association has been shown in disabled or chronically ill children after PEG placement. Our results showed that slightly over half of the hospitalizations in this population were due to respiratory symptoms. Due to the small number of patients with confirmed vitamin D deficiency, the association between vitamin D levels and the incidence of hospitalization due to respiratory symptoms was not statistically significant in our cohort. Previous studies have shown an improvement in the nutritional status after PEG insertion, particularly in anthropometric measurement [1,29,32,33,34]. Data regarding micronutrient deficiencies in this group of patients are limited. Despite our inability to evaluate anthropometric outcomes, except for weight z score (such as height, skinfold thickness, or mid-arm circumference) based on our results, routine laboratory work-up for micronutrient levels should be considered, particularly vitamin D, which may reduce hospitalization rates due to respiratory infections, although we were unable to demonstrate this association or causality in our study. This needs to be assessed further in prospective observational studies, particularly in those who are not on exclusive GT feeding.

In our research, a small percentage of children underwent fundoplication after GT insertion, a percentage that is similar to previously published numbers over the last two decades [13], but much higher than ~1% of previously published cohorts from Boston Children’s Hospital [35]. This difference can be explained by a longer follow-up period and a population that is more diverse with different approaches among several medical centers in our study. In our cohort, one-fifth of the patients underwent a second anti-reflux procedure following the initial fundoplication, consistent with findings from previous studies [36,37].

Our study has allowed us to identify several factors associated with subsequent fundoplication and recurrent hospitalizations due to respiratory tract infections. Patients treated with proton pump inhibitors (PPIs) or inhaled corticosteroids/beta-blockers constitute a significantly higher-risk group that should benefit from a reinforced close follow-up and assessments. The association between PPI use and fundoplication is most likely due to the presence of non-PPI-responsive gastroesophageal reflux disease (GERD) or esophagitis, which necessitated surgical intervention. According to our knowledge, no previous cohorts over the last two decades evaluated these outcomes among children after PEG insertion.

Our study has several strengths, including the relatively large number of patients treated in several hospitals by different teams and protocols, as well as the prolonged follow-up of more than a decade. However, our study was limited by its retrospective nature and being a database study, which limited complete data collection, including family socioeconomic status, anthropometric measurements (except for weight z-scores), use of vitamin and iron supplements, laxative and PPI doses and treatment durations, comprehensive micronutrient assessments, and complications related to GT insertion. Another limitation was that around 10% of the patients were lost to follow-up, and moved to another HMO in Israel, which may have led us to underestimate the frequency of complications. Furthermore, we had limited data on the proportion of patients on exclusive GT feeding, in whom the likelihood of micronutrient deficiencies, including vitamin D or iron, is low. Finally, we did not have data regarding the diagnostic workup for GERD, such as barium studies or pH-impedance testing. We recognize these limitations and suggest future standardized documentation studies to address these issues. Despite these limitations, this study included one of the largest pediatric cohorts to date, with a relatively long follow-up and specific measures for outcomes and disease course.

## 5. Conclusions

In conclusion, this study confirmed the durability of gastrostomy tube feeding among children with neurological impairment and the relatively low prevalence of fundoplication over a long period of follow-up. Many patients had frequent hospitalizations, primarily due to respiratory infections. We demonstrated a high prevalence of vitamin D deficiency among children whose vitamin D status was assessed. However, the association between vitamin D deficiency and admission due to respiratory infections was not shown. Our findings underscore the importance of integrating regular micronutrient monitoring into clinical protocols for gastrostomy-fed children. Such measures could mitigate complications, improve long-term outcomes, and optimize individualized nutritional management in this vulnerable population. Physicians following gastrostomy-fed children with neurological impairment might consider more frequent assessments of micronutrient deficiencies, particularly vitamin D.

## Figures and Tables

**Table 1:** Patients’ characteristics.

Variables	
Female Gender, Number (%)	184 (49)
Age at GT insertion, Median (IQR) Months	22.8 (6.5–47.2)
Type of Formula, Number (%)	
Polymeric	348 (92)
Extensively hydrolyzed or free amino acid	30 (8)
Diagnosis, Number (%)	
Neurodevelopmental delay	327 (88)
Seizures	82 (22)
Kidney Disease	12 (3)
Malignancy	3 (1)
Indication for GT insertion, Number (%)	
Failure to thrive	141 (38)
Recurrent pneumonia	106 (29)
Recurrent wheezing	184 (49)
Recurrent vomiting	198 (53)
Feeding difficulties	275 (74)

**Table 2:** Predictors of recurrent hospitalizations due to respiratory infections.

Variables	No Recurrent Respiratory Hospitalizations, *n* = 252	Recurrent Respiratory Hospitalizations, *n* = 120	HR (95% CI)	*p*	Adjusted * HR (95% CI)	*p*
Male *n*, (%)	121 (48)	74 (62)	2.26 (1.11–4.8)	0.038	1.5 (0.72–2.86)	0.188
Neurodevelopmental delay *n*, (%)	214 (85)	114 (95)	1.47 (1.105–2.77)	0.043	1.15 (0.88–1.38	0.101
Seizures *n*, (%)	30 (12)	52 (43)	2.45 (0.618–1.88)	0.002	1.502 (1.302–6.642)	0.014
Jewish ethnicity *n*, (%)	58 (23)	24 (20)	1.942 (0.67–3.117)	0.221		
Age at GT insertion—median (IQR) Months	30.1 (16.5–44)	21.8 (11.6–30.9)	0.928 (0.882–0.992)	0.041	0.901 (0.586–1.427)	0.259
PPIs therapy *n*, (%)	125 (50)	72 (60)	2.041 (1.442–3.86)	0.021	1.88 (1.46–3.07)	0.033
Inhaled b-blockers/corticosteroids therapy *n*, (%)	106 (42)	83 (69)	1.508 (1.246–2.49)	0.001	1.45 (1.28–3.25)	0.003
Antibiotic prophylaxis *n*, (%)	30 (12)	18 (15)	1.26 (0.93–2.52)	0.089	1.22 (0.88–1.556)	0.24
Iron deficiency *n*, (%)	149 (59)	77 (64)	1.28 (0.62–2.07)	0.445	1.128 (0.627–3.35)	0.195
VIT D deficiency *n*, (%)	14 (5.5)	11 (9.2)	2.24 (0.92–3.52)	0.102	1.562 (0.952–1.98)	0.216

Adjusted * HR: means for multivariate analysis.

**Table 3:** Predictors of fundoplication during follow-up.

Variables	No Fundoplication, *n* = 327	Underwent Fundoplication, *n* = 45	HR (95% CI)	*p*	Adjusted * HR (95% CI)	*p*
Male *n*, (%)	171 (52)	24 (53)	2.03 (1.01–4.1)	0.048	1.9 (0.76–4.76)	0.171
Neurodevelopmental delay *n*, (%)	283 (87)	44 (98)	1.67 (0.705–3.97)	0.243		
Seizures *n*, (%)	74 (23)	8 (18)	1.15 (0.88–1.38)	0.127		
Jewish ethnicity *n*, (%)	72 (22)	10 (22)	1.82 (0.77–2.97)	0.422		
Age at GT insertion—median (IQR) Months	27 (14.5–52)	23.4 (11.6–34.9)	1.05 (1.03–1.08)	0.021	0.95 (0.66–1.37)	0.779
PPIs therapy *n*, (%)	155 (47)	42 (93)	2.81 (1.32–4.62)	<0.001	1.95 (1.66–2.37)	<0.001
Recurrent vomiting during follow-up *n*, (%)	77 (24)	15 (33)	1.7 (0.93–2.8)	0.089	1.8 (0.56–3.67)	0.782
Inhaled b-blockers/corticosteroids therapy *n*, (%)	153 (47)	36 (80)	1.46 (1.36–1.93)	0.012	1.25 (1.1–3.05)	0.041
Antibiotic prophylaxis *n*, (%)	39 (12)	9 (20)	1.97 (1.63–2.2)	0.039	1.8 (0.86–2.46)	0.222
Frequent recurrent hospitalizations during follow-up *n*, (%)	194 (59)	36 (80)	1.88 (1.71–3.27)	0.043	2.15 (0.86–2.76)	0.101
Iron deficiency *n*, (%)	199 (70)	27 (71)	1.65 (0.42–3.7)	0.414	1.13 (0.37–3.5)	0.835

Adjusted * HR: means for multivariate analysis.

## Data Availability

The data presented in this study are available on request from the corresponding author, due to ethical reasons.
